# COVID-19 Coagulopathy

**DOI:** 10.3390/life14080953

**Published:** 2024-07-29

**Authors:** Andrew Rettew, Ian Garrahy, Shoja Rahimian, Rebecca Brown, Navdeep Sangha

**Affiliations:** Tower Health System, Reading Hospital, West Reading, PA 19611, USA; andrew.rettew@towerhealth.org (A.R.); shoja.rahimian@towerhealth.org (S.R.); rebecca.brown@towerhealth.org (R.B.); navdeep.sangha@towerhealth.org (N.S.)

**Keywords:** coagulopathy, SARS-CoV-2, vasculopathy

## Abstract

Coronavirus disease of 2019 (COVID-19) is the respiratory viral infection caused by the severe acute respiratory syndrome coronavirus 2 (SARS-CoV-2). Despite being a primary respiratory illness, it is commonly complicated by systemic involvement of the vasculature leading to arterial and venous thrombosis. In this review, we will focus on the association between COVID-19 and thrombosis. We will highlight the pathophysiology of COVID-19 coagulopathy. The clinical manifestations of COVID-19 vasculopathy will be discussed with a focus on venous and arterial thromboembolic events. COVID-19 vasculopathy and disseminated intravascular coagulation (DIC) are distinguished within, as well as areas of controversy, such as “long COVID”. Finally, the current professional guidelines on prevention and treatment of thrombosis associated with SARS-CoV-2 infection will be discussed.

## 1. Introduction

Coronavirus disease of 2019 (COVID-19) is caused by the severe acute respiratory syndrome coronavirus 2 (SARS-CoV-2). The Word Health Organization (WHO) declared COVID-19 a pandemic in March 2020. COVID-19 is a primarily respiratory illness that can lead to pneumonia or acute respiratory distress syndrome (ARDS). Despite being a primary respiratory illness, complications via systemic involvement of the vasculature can arise. Vascular involvement leads to an array of complications ranging from venous and arterial thrombosis to pulmonary edema secondary to loss of barrier function. 

Venous and arterial thrombosis have been extensively reported in patients infected with SARS-CoV-2. Various organs are affected by COVID-19 vasculopathy, including the vasculature of lungs [1], legs [2], spleen [3], heart [4], and brain [5]. These thromboembolic complications are often associated with multiorgan failure and high morbidity and mortality. The most frequently noted thrombotic events in COVID-19 are deep vein thrombosis (DVT) and pulmonary embolism (PE) [6]. The risk of venous thromboembolism (VTE) is high in hospitalized patients despite anticoagulation prophylaxis, with a 20–69% DVT/PE rate reported [7,8,9,10,11]. Other vascular clinical manifestations include stroke, acute limb ischemia, and myocardial infarction [5,12,13]. This review will summarize the mechanisms by which SARS-CoV-2 infection can lead to thromboembolic events.

### 1.1. Pathophysiology of COVID-19 Coagulopathy

**Figure 1:**
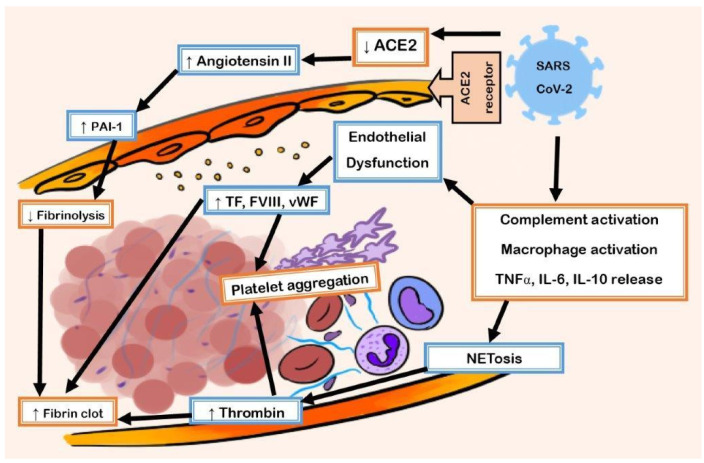
Pathophysiology of the association between thrombosis and COVID-19. SARS-CoV2 binds to the ACE2 receptor. ACE2 converts angiotensin II to angiotensin 1-7. SARS-CoV-2 entry into the cell leads to reduced ACE2 activity. This leads to an increase in Angiotensin II signaling. SARS-CoV2 initiates the release of inflammatory cytokines resulting in activation of neutrophils and macrophages. The endothelium is damaged due to proinflammatory signals. This exposes the prothrombotic basement membrane. It also leads to increased tissue factor, factor VII, and vWF, which leads to activation of platelets and the coagulation cascade. Inhibition of PAI-I leads to suppressions of fibrinolysis. Further amplifying the prothrombotic state is the crosstalk between the immune, complement, and coagulation systems. SARS-CoV2, *severe acute respiratory syndrome coronavirus 2*; ACE2, *angiotensin converting enzyme 2*; NET, *neutrophil extracellular trap*; vWF, *von Willebrand factor*; WPB, *Weibel Palade body*; PAI-1, *plasminogen activator inhibitor 1*.

There can be significant sequelae resulting from SARS-CoV-2 reaching the respiratory system. When SARS-CoV-2 binds the ACE2 receptor, there is an inflammatory cascade involving cytokines, white blood cells, and the complement system leading to fibrinolysis suppression and endothelial dysfunction (Figure 1). This cascade leads to the coagulopathy that results in COVID-19.

### 1.2. Entry of SARS-CoV-2 in ACE2

SARS-CoV-2 binds to the transmembrane angiotensin-converting enzyme 2 (ACE2) receptor via its spike protein. ACE2 is expressed ubiquitously, including in lung alveolar pneumocytes, as well as in endothelial cells, the heart, and the kidneys [12]. ACE2 plays a critical role as a negative regulator of the renin-angiotensin-aldosterone system (RAAS). It does so by inactivating the pro-inflammatory angiotensin II by converting it to angiotensin 1-7 [13]. 

When SARS-CoV-2 binds to the ACE2 receptor, it initiates a cascade that ultimately leads to the downregulation of the receptor [14]. Whereas Angiotensin II is a vasoconstrictor with prothrombotic effects, Angiotensin 1-7 is a vasodilator. Angiotensin 1-7 is a vasodilator as a result of increased production of nitric oxide and prostacyclin. Increasing production of these vasodilatory molecules suppresses platelet activation [15]. Downregulation of ACE2 leads to unopposed angiotensin II effects, causing proinflammatory and prothrombotic changes. As such, uncontrolled RAAS in the endothelium of COVID-19 patients is a main reason for the cascade that leads to coagulopathy [16]. 

### 1.3. Hyperinflammatory Response and Oxidative Stress Damage

With regular breathing, alveolar macrophages and neutrophils provide immune surveillance in the alveoli through signaling pathways that inhibit proinflammatory pathways mediated by NF-kB, MAPK, STAT3, and MyD88 [17]. This constant surveillance allows for the clearance of infectious pathogens that the alveolar-capillary unit meets with regular breathing. 

The respiratory endothelium reacts to the inflammatory cascade of SARS-CoV-2 infection via several receptors and intracellular signaling pathways. The release of cytokines is stimulated by pathogen-associated molecular patterns (PAMPs) and host derived damage-associated molecular patterns (DAMPs), and ultimately leads to a hyperinflammatory response [18,19]. The release of cytokines from monocytes, macrophages, and neutrophils activates the proinflammatory pathways mediated by NF-kB, MAPK, STAT3, and MyD88 [17]. Such cytokines that are released in the setting of SARS-CoV-2 infection include IL-6, IL-10, and tumor necrosis factor alpha (TNF-α) [20]. 

As aforementioned, ACE2 leads to the conversion of angiotensin II to angiotensin 1-7. SARS-CoV-2 entry into the cell leads to reduced ACE2 activity. Downregulation of ACE2 leads to increased angiotensin II and decreased angiotensin 1-7. Increased angiotensin II leads to vasoconstriction, but also oxidative stress damage, which is mediated by nicotinamide adenine dinucleotide phosphate (NADPH) activation and the production of reactive oxygen species (ROS). Conversely, angiotensin 1-7 removes ROS by increasing production and release of nitric oxide from endothelial cells [15]. The inflammatory cascade of SARS-CoV-2 infection results in increased generation of ROS by activation of NADPH and decreasing nitric oxide production from endothelial cells. These changes to the endothelium lead to a transformation from clearance of an infectious pathogen to a hyperinflammatory response. 

The results of this hyperinflammatory response on the endothelium are significant. It leads to prothrombotic changes and the loss of barrier function. The loss of barrier function results in capillary leak and alveolar hemorrhage [21].

### 1.4. Prothrombotic Transformation of Endothelium

Thrombomodulin (TM) is a glycoprotein on the surface of endothelial cells that serves as a receptor for thrombin (IIa). It blocks the fibrin binding site of thrombin. TM is a natural anticoagulant since it functions as a cofactor in the thrombin-induced activation of protein C, resulting in activated protein C (APC). APC is a strong anticoagulant that inactivates clotting factors Va and VIIIa. The endothelium expresses tissue factor pathway inhibitor-β (TFPIβ). TFPIβ inhibits blood coagulation by binding and inhibiting factor VIIa (FVIIa) and factor Xa (FXa), which results in the inability of tissue factor (TF) to initiate coagulation. The primary function of plasmin is to clear fibrin. The endothelium secretes tissue plasminogen activator (tPA) and urokinase plasminogen activation (uPA), which convert the zymogen plasminogen to plasmin, initiating the process of fibrinolysis. Plasminogen activator inhibitors 1 (PAI-1) inhibits tPA and uPA. As such, PAI-I is an inhibitor of fibrinolysis. 

The upregulation of TF on monocytes and macrophages stimulated by TNF-α, IL-1β, IL-6, and the downregulation of TM results in the activation of clotting factor Xa. This activates the extrinsic pathway of the coagulation cascade [22]. Externalization of plasma membrane phosphatidylserine promotes the prothrombinase complex, leading to the activation of factor IIa (thrombin). There is suppression of fibrinolysis with the activation of Thrombin Activable Fibrinolysis Inhibitor (TAFI) and increased release of PAI-1 from endothelial cells and activated platelets secondary to the hyperinflammatory response [23]. Increased PAI-1 and TAFI expression are risk factors for thrombosis given the resulting fibrinolysis suppression. 

### 1.5. Role of the Complement System

The complement system plays a significant role in the innate immune response. It also has significant crosstalk with the coagulation system. The complement system can activate the coagulation cascade in multiple ways. Complement factor C3 and the membrane attack complex (MAC) directly lead to platelet activation and aggregation [24]. Complement factor C5a increases the expression of PAI-1 [25]. Both mechanisms ultimately lead to thrombosis.

SARS-CoV-2 entry into the cell leads to activation of the complement system. MAC causes the endothelium to secrete vWF and facilitates the assembly of the prothrombinase complex. High complement levels have been associated with microvascular thrombosis on autopsy findings in patients with severe COVID-19 infection. High C3a and C5a levels on admission are associated with severe infection, as are decreases in serum C3 and C4 [26].

The evidence supporting the use of complement inhibition in SARS-CoV-2 infection has been mixed. The anti-C5 monoclonal antibody eculizumab has been used in COVID-19 patients [27], but a study evaluating the longer-acting anti-C5 monoclonal antibody ravulizumab was discontinued early due to lack of efficacy. 

### 1.6. Neutrophil Extracellular Traps (NETs)

Neutrophils also play an integral role in the innate immune response. As neutrophils reach their infectious targets, they release extracellular strands of chromatin, which contain antimicrobial proteins. These neutrophil extracellular traps (NETs) capture and kill pathogens to prevent the dissemination of infectious particles. Despite being necessary for the innate immune response, excessive NETs (NETosis) formation can be harmful to the host. It appears that NETosis contributes to increased risk of thrombosis with COVID-19 [28]. There are several mechanisms proposed by which NETs interact with the coagulation system, thereby leading to thrombosis. Activated neutrophils express TF, which activates the intrinsic pathway of the coagulation cascade. The prothrombotic effect of NETs is highlighted by crosstalk between the endothelium, platelets, and neutrophils, resulting in a vicious hypercoagulable cycle [29].

### 1.7. Fibrinolysis Suppression

Suppressed fibrinolysis due to elevated levels of PAI-1 has been described in acute lung injury and sepsis [30]. This knowledge led to the theory that the fibrinolytic system may play an important role in acute lung injury caused by SARS-CoV2 infection.

Suppressed fibrinolysis has been reported in SARS-CoV2 infected patients, which was mainly associated with elevated PAI-1 levels [23]. Furthermore, fibrinolysis shutdown was reported in a large proportion of COVID-19 ICU patients via thromboelastography testing, and these patients had a higher rate of thrombotic complications [31].

Several mechanisms have been proposed to explain suppression of fibrinolysis in COVID-19. One mechanism is the high levels of thrombin activable fibrinolysis inhibitor (TAFI) and the release of PAI-1 from endothelial cells [23]. A second mechanism is dysregulated RAAS due to SARS-CoV-2 mediated downregulation of ACE2, leading to unopposed Angiotensin II and increased levels of PAI-1 [6]. 

### 1.8. Endothelial Dysfunction

The critical endothelium barrier between the blood compartment and the extracellular space is compromised following severe SARS-CoV-2 infection. The alveolar-capillary interface regulates gas exchange, performs essential barrier functions, maintains blood flow and hemostasis, and controls leukocyte trafficking. The resting endothelium promotes nitric oxide and prostacyclin production, which inhibits platelet activation. Conversely, inflamed endothelium promotes platelet activation. Severe SARS-CoV-2 infection leads to a reduction in the release of nitric oxide and prostacyclin, thereby leading to platelet activation, adhesion, and aggregation, and to thrombotic complications. 

Inflammatory cytokines, PAMPs, and DAMPs stimulate the endothelium to release vWF from Weibel-Palade bodies in the setting of systemic SARS-CoV-2 infection. vWF binds to GPIbα on platelets, and to endothelial α_v_β_3_, which in turn binds fibrinogen. These mechanisms facilitate the adhesion of platelets to activated endothelium and contribute to microvascular occlusion, as has been observed in autopsy specimens of lungs from patients who died of COVID-19 [32]. This suggests that elevated levels of vWF can serve as a biomarker predictive of adverse outcomes in severe COVID-19 [20]

As aforementioned, constitutive signaling through cytoprotective Tie2 signaling serves to provide for the clearance of microbes that the alveolar-capillary functional unit encounters. When there is thrombosis as a complication of severe COVID-19 infection, loss of laminar flow and high shear stress from the thrombosis inhibit Tie2 signaling. This further suppresses the capacity of the endothelium to vasodilate by inhibiting nitric oxide synthesis [33]. The unopposed Angiotensin II resulting from SARS-CoV-2 mediated downregulation of ACE2 leads to compromise of the endothelium and eventual and endothelial cell death.

## 2. Biomarkers in COVID-19 Coagulopathy

Coagulopathy is a commonly recognized feature of the inflammatory response in COVID-19, as 20–50% of hospitalized patients are noted to have abnormalities with elevated D-dimer, prolonged prothrombin time, thrombocytopenia, and abnormal fibrinogen levels. While some of these values resemble DIC or the coagulopathy seen with sepsis, there are clear differences in the patterns of laboratory abnormalities as well as the clinical complications [34]. The coagulopathy tests of COVID-19 often result in either mild DIC or non-diagnosis of DIC when applied to the DIC scoring system of the International Society on Thrombosis and Haemostasis (ISTH) [35]. The related thrombocytopenia and prolonged prothrombin time tend to be milder than that of DIC. Also, fibrinogen tends to be normal or elevated as an acute phase response rather than reduced in contrast to frank DIC. Thus, COVID-19-associated coagulopathy (CAC) has been separated into its own clinical entity with a unique definition and criteria, as suggested by Iba et al. for better delineation in comparison to the ISTH criteria for DIC (Table 1) [36]. 

The commonly elevated D-dimer in CAC may be one of the only similarities among laboratory findings of DIC and CAC. The D-dimer with COVID-19 results initially from coagulation activation in the pulmonary microvasculature. D-dimer is also reflective of the highly inflammatory disease state and can vary with the more common occurrence of thrombosis as opposed to bleeding. In one study, patients without thrombotic or bleeding complications had a median D-dimer range of 760 to 1377 ng/mL, while those with thrombosis had values of 1336–4001 ng/mL, and 928–3625 ng/mL in those with bleeding. The peak D-dimer values correlated most strongly to the C-reactive protein, the erythrocyte sedimentation rate, and to procalcitonin [37]. D-dimer also correlates to patient outcomes in COVID-19, where it has been noted that an elevated D-dimer increases risk of death fourfold [38].

While DIC can have both thrombotic and bleeding phenotypes, the uncommonness of bleeding with CAC presents a key clinical difference between the two [39]. Bleeding has been reported to occur in less than 5% of hospitalized patients with COVID-19, although those with severe respiratory compromise treated with extracorporeal membrane oxygenation have been noted to have higher rates of up to 12%. The lesser risk for bleeding complications with CAC may be related to the suppression of fibrinolysis, particularly in earlier disease, where the coagulation activation and the microthrombi are limited to the lungs rather than the more systemic DIC [40].

The development of frank DIC has been mostly noted in severe stages of or in non-survivors of COVID-19. In one early evaluation of COVID-19 non-survivors, 71.4% were identified as having developed DIC by ISTH criteria, as further increases in D-dimer and PT prolongation as well as decreases in fibrinogen were noted. Only 0.6% of the survivors had DIC [41].

## 3. Clinical Manifestations of COVID-19 Vasculopathy

### 3.1. Venous Thromboembolism

Although the damage sustained to systemic endothelium by SARS-CoV-2 can cause a wide array of clinical sequelae, thrombotic complications are by far the most common. The incidence of thrombosis among COVID-19 patients has been reported in a wide range, ranging from around 10% to 30% in various studies performed at multiple institutions since the spring of 2020. In a review study published in August 2020, about half of all patients presenting with COVID-19 experienced a thrombotic event within 24 h of admission [42]. With the limited knowledge of the pathophysiology of COVID-19 vasculopathy in the infancy stages of the pandemic, SARS-CoV-2 related thrombosis was a troubling issue for clinicians due to the increased rates of associated morbidity and mortality. 

Venous thromboembolism (VTE) has emerged as the most common form of COVID-19 associated thrombosis [43]. DVT and PE were frequently reported complications of hospitalized COVID-19 patients, especially patients admitted to the ICU. One multicenter study performed in the United States suggested that COVID-19 patients admitted to the ICU were more than twice as likely to develop VTE [44]. PE was likely the most frequent thrombotic complication of SARS-CoV-2 regardless of clinical detection prior to discharge or death (Angelini). In one study, most patients were found to have numerous pulmonary microthrombi on autopsy, and about 20% of the patients were found to have major pulmonary thrombi resulting in infarcts or hemorrhage [45]. Clinically, massive and submassive PE was a common cause of death among hospitalized COVID-19 patients despite the use of thromboprophylaxis in early 2020 [46]. 

DVT was another common clinical manifestation of COVID hypercoagulability, although the incidence of DVT in these critically ill patients was likely confounded by the length of ICU stay, immobilization, and severe sepsis or septic shock. However, the embolic relationship between DVT and PE in these patients was not as clearly defined, and many patients who developed PE were not found to have a concurrent DVT on ultrasound imaging [47]. This provides further evidence for the development of thrombi based on local endothelial damage and inflammatory cascades, which promoted the formation of local thrombi. As described above, the significant damage sustained to alveolar membranes and pulmonary vasculature by viral mechanisms propagated an overwhelming response which in turn caused the in-situ formation of thrombi in the pulmonary vasculature, which is not commonly seen in other disease processes. Overall, VTE of any type was associated with a significant increase in mortality; an American study performed in August of 2020 suggested an almost 75% increased risk of mortality in COVID-19 patients who developed any type of VTE [48]. 

Recent studies have shown a decrease in the incidence in VTEs in hospitalized patients with COVID-19 [49]. Interpreting this data has been challenging due to the extrinsic factors contributing to these statistics over the course of this global pandemic. Most studies considering thrombosis in COVID-19 patients were performed on patients who were hospitalized due to the severity of their disease, which may exclude a significant population of patients who may have experienced thrombosis while being treated on an outpatient basis [50]. Management with prophylactic anticoagulation strategies may have played a role in preventing episodes of thrombosis as they were developed over the course of the past three years [51]. Use of specific diagnostic modalities such as ultrasound and CT imaging were clearly superior in the detection of thromboses, but may not have been used by clinicians initially due to the unfamiliarity with the significant hypercoagulability of this disease [52]. Finally, thrombosis data from these studies may also have been influenced by the advent of the novel SARS-CoV-2 vaccine that became available in late December 2020. High rates of vaccination and the subsequent prevention of severe COVID-19 requiring inpatient admission may have contributed to the seeming decline in the incidence of thrombosis in more recent meta-analyses [53]. In contrast, early data published regarding rates of thrombosis prior to vaccine availability may have been grossly underestimated without the consistent use of diagnostic modalities, prophylactic anticoagulation, and the inclusion of non-hospitalized patients [8]. 

### 3.2. Arterial Thrombotic Events

Although venous thromboembolism was the most common (and perhaps most easily detected) manifestation of coagulopathy among COVID-19 patients, arterial thrombotic events were frequently reported. As with COVID-19 associated VTE, arterial thromboemboli were more common in critically ill patients, but were more anatomically diverse [7]. For example, arterial thrombus formation was noted commonly in limb arteries leading to limb ischemia, but was also a common cause of stroke when formation occurred in cerebral arteries [54]. Also documented over several studies were mesenteric ischemia caused by the formation of thrombi in the superior mesenteric artery, myocardial infarction caused by thrombi in the coronary arteries, and even the formation of thrombi within large vessels such as the aorta, carotids, and iliac arteries. These events were typically associated with a significant symptom burden in an overwhelming majority of the patients (over 95%) and with significant mortality [55,56]. 

A particularly alarming aspect of these arterial thromboembolic events was a considerable delay in diagnosis due to the critically ill nature of the affected patients. Because many COVID-19 patients required prolonged mechanical ventilation, the typical symptoms associated with stroke, myocardial infarction, or limb ischemia were unable to be communicated to clinical staff and therefore remained undetected. Eventually, derangements in vital signs or clinical symptoms such as skin changes or restlessness would prompt diagnostic studies to be performed, but likely days after an embolic event had occurred. At the time of diagnosis, the typical interventions used to prevent tissue loss were unable to be performed [57]. Although less common than venous thromboembolic events, arterial thromboembolic events were a significant cause of morbidity and mortality in COVID-19 patients, perhaps due in part to the delay in their diagnosis in the ICU setting.

### 3.3. Long COVID-19

A significant number of patients report persistent or new symptoms despite the clearance of SARS-CoV-2 infection. The most common symptoms reported include malaise, weakness, fatigue, concentration impairment, headache, and shortness of breath [58]. Although the cause of these symptoms are poorly understood, data are emerging which suggest that vasculopathy may persist after clearance of the viral infection. Some studies show long term fibrotic changes on computed tomography and abnormal pulmonary function testing, particularly with the diffusing capacity for carbon monoxide [59,60]. Elevated D-dimer levels have been found in plasma from convalescent patients, and continued immune activation suggests ongoing endothelial dysfunction [61]. Nevertheless, 90-day follow-ups for thrombotic complications in those with hospitalizations has not identified higher rates of venous thromboembolism upon post-hospitalization. The rates in one study of 129 patients treated in the ICU had similar rates of inpatient venous thrombosis to typical hospital-associated rates, however there were no events recorded after discharge to outpatient [62]. 

## 4. Management of COVID-19 Related Venous Thromboembolism

### 4.1. Current Practice Guideline Recommendations for Prophylaxis of VTE in Hospitalized Patients with COVID-19

Numerous professional societies have published guidelines on VTE prophylaxis and VTE treatment in patients with COVID-19. Much of the available data stems from trials attempting to elucidate the clinical benefit to antithrombotic therapy in various COVID-19 patient populations, namely the REMAP-CAP, ACTIV-4a, and ATTACC trials (ClinicalTrials.gov numbers, NCT02735707, NCT04505774, NCT04359277, and NCT04372589). These multi-platform, randomized control trials investigated the benefit of antithrombotic therapies, ranging from standard, prophylactic dose heparin products to therapeutic dose regimens in both the critically ill and non-critically ill patient populations.

In a meta-analysis of all three trials, patients were randomly assigned to receive therapeutic-dose heparin anticoagulation or thromboprophylactic-dosing with either unfractionated (UH) or low molecular weight heparin (LMWH). The primary outcome was measured in organ-support free days. In the critically ill cohort, the survival to hospital discharge ratio was not statistically different (65 vs. 63 percent; odds ratio 0.84, 95% CI 0.64–1.11) between the two dose intensities [63,64]. Additionally, the therapeutic-dose arm of the study was found to have a 1.9%, non-statistically significant higher bleeding risk. Most of the patients (71%) in the thromboprophylaxis arm of the trial received a low dose thromboprophylactic drug, while 26% received an intermediate dose. This likely abrogated closer examination of the differences between low and intermediate thromboprophylactic dosing in the prophylaxis arm. No benefit was appreciated between intermediate-dose and standard-dose prophylactic anticoagulation in reducing the risk of death, treatment with ECMO, or venous or arterial thrombosis [65]. As such, the consensus amongst the different published guidelines is to employ prophylactic dose heparin products for VTE prophylaxis in critically ill hospitalized patients. 

In contrast, the non-critically ill patient arm of the ACTIV-4a, REMAP-CAP, and ATTACC trials did show a 4% difference in survival until hospital discharge without organ support, favoring the therapeutic-dose arm (adjusted OR 1.27, CI 1.03 to 1.58). There was a 1%, non-statistically significant increase in bleeding risk with therapeutic dosing (1.9% vs. 0.9% in the prophylactic-dose arm) [63,64]. This data was further corroborated by the HEP-COVID trial, which showed the benefit of full dose anticoagulation in non-critically ill patients with D-dimer levels elevated at least 4 times above the upper limit of normal, and which showed no benefit in the critically ill patient population [66]. The anticoagulation forum recommends therapeutic-dose heparin products for VTE prophylaxis in non-critically ill hospitalized patients. 

The correlation between D-dimer, the anticoagulation strategy, and thrombosis was also examined in the ACTION trial completed in Brazil. In this trial, investigators attempted to determine the benefit of therapeutic vs. prophylactic dose anticoagulation in all patients with COVID-19 infection and elevated D-dimer. Patients were randomly assigned to either dosing regimen at the time of hospitalization and were continued on anticoagulant therapy until 30 days post discharge. The primary outcome was time to death, duration of hospitalization, or duration of supplemental oxygen to day 30. The primary safety outcome was major or clinically relevant, non-major bleeding through the 30 days. The study revealed no difference in primary outcome regardless of the dosing regimen or the clinical illness severity. There was, however, a statistically significant increased risk of bleeding (relative risk 3.64 [95% CI 1.61–8.27], *p* = 0.0010) [67].

### 4.2. Recommendations for Prophylaxis of VTE in Non-Hospitalized Patients with COVID-19

COVID-19 infected patients who were not hospitalized were also thought to be at a higher risk for thrombosis. The ACTIV-4b trial attempted to determine the risk reduction benefits from aspirin, thromboprophylaxis-dose apixaban, or therapeutic-dose apixaban in the outpatient patient population. This trial was also shuttered early due to low incidence of primary outcome events (all-cause mortality, symptomatic venous or arterial thromboembolism, myocardial infarction, stroke, or hospitalization for cardiovascular or pulmonary causes). As a composite, these events occurred in 0.0% of the aspirin group, 0.7% of the prophylactic apixaban group, 1.4% of the full dose apixaban group, and 0.0% of the placebo group, respectively; there were no significant differences between the active groups and the placebo group. This data failed to show any benefit to antiplatelet or anti-thrombotic therapy in outpatients with COVID-19 infection [68]. The only guideline to address outpatient management of COVID-19, the Global COVID-19 Thrombosis collaborative Group, recommends increased mobility with the consideration of anticoagulant prophylaxis for those with limited mobility, history of VTE, or active malignancy [65].

### 4.3. Recommendations for Extended Duration Prophylaxis of VTE in Hospitalized Patients with COVID-19

Another group of interest in the outpatient setting were those who had recently been discharged from the hospital after a COVID-19 related inpatient admission. The Brazilian MICHELLE trial investigated the benefit of continued post discharge prophylactic-dose anticoagulation in those patients with high risk for venous thromboembolism, defined as those with an IMPROVE VTE score >4 or >2 and with a D-dimer over 500 ng/mL. In this randomized control trial, the primary outcome measured was fatal, symptomatic, or asymptomatic VTE, and cardiovascular death at 35 days post discharge. This trial did show an absolute risk reduction of 6.2% (relative risk 0.33, 95% CI 0.12–0.90; *p* = 0.0293) for those receiving thromboprophylaxis after discharge without any major bleeding events in either arm [69]. 

Extended duration VTE prophylaxis can be considered for patients who are at a low bleeding risk and who were previously admitted to the ICU, intubated and sedated for multiple days, or who have ongoing VTE risk factors at the time of hospital discharge. Though the benefit of extended duration VTE prophylaxis remains under examination, it is reasonable to treat this higher risk population (as stratified by the IMPROVE VTE score) with LMWH or a DOAC for up to 45 days. 

### 4.4. Current Practice Guideline Recommendations for Treatment of VTE in Patients with COVID-19

For treatment of VTE in hospitalized patients with COVID-19, most guidelines suggest parenteral anticoagulation with a transition to a DOAC as the patient transitions to the outpatient setting [66,70]. When using therapeutic-dosed unfractionated heparin, the guidelines suggest monitoring anti-Xa levels rather than aPTT, as prolonged aPTT with elevated levels of factor VIII and positive lupus anticoagulants is common [71].

Various international societies have subsequently published clinical practice guidelines regarding the management of venous thromboembolism prophylaxis in the COVID-19 patient population; these are summarized below (Table 2). It should be noted that, in general, there appears to be consensus to avoid asymptomatic screening for venous thrombosis, and to continue or initiate anticoagulation at appropriate doses for non-COVID-19 related indications, with attention to their usual standard of care. Most guidelines recommend LMWH over UFH for prophylaxis, primarily to decrease the number of injections, risk of healthcare provider exposure, and risk of heparin-induced thrombocytopenia (HIT). 

## Figures and Tables

**Table 1:** Comparison of coagulopathy criteria between CAC and DIC.

Proposed CAC Criteria: ≥2 of the following:		ISTH DIC Criteria: ≥5 Points
D-dimer	>2x ULN	<5x ULN = 2, ≥5x ULN= 3
Platelet count	<150 × 10^9^/L	<100 = 1, <50 = 2
Prolonged PT	>1 s or INR > 1.2	≥3 s to <6 s = 1, ≥6 s = 2
Thrombosis	Presence, micro or macrovascular	Not part of the criteria
Fibrinogen	Not part of the criteria, but considered at risk for CAC if elevated	≤1.0 g/L = 1
	Other risk factors: vWF > 2x ULN, presence of high-titer antiphospholipid antibodies and lupus anticoagulant	

ULN, upper limit of normal; PT, prothrombin time; INR, international normalized ratio; vWF, von Willebrand factor.

**Table 2:** Current practice guidelines for prophylaxis of VTE in patients with COVID-19.

Society/Guideline	Outpatient Prophylaxis	Non-Critically Ill Prophylaxis	Critically Ill Prophylaxis	Post Discharge Prophylaxis
ASH	No recommendation	Therapeutic dose	Prophylactic dose	Yes
ISTH	No recommendation	Prophylactic dose Or intermediate dose	Prophylactic dose Or intermediate dose	In high-risk patients
Chest 2020	No recommendation	Prophylactic dose	Prophylactic dose	No recommendation
NIH	No recommendation	Therapeutic dose if elevated d-dimer and on low flow oxygen	Prophylactic dose	Routine use not recommended
Anticoagulation Forum	No recommendation	Prophylactic dose, consider therapeutic in high-risk patients	Prophylactic dose	In high-risk patients
